# Surgical Salvage of Annular Rupture After Transcatheter Aortic Valve Implantation by Conversion Into Annular Enlargement

**DOI:** 10.1016/j.jaccas.2022.08.014

**Published:** 2022-11-16

**Authors:** Lamees I. El Nihum, Qasim Al Abri, Amr Telmesani, Areeba Ali, Yuncen A. He, Tomoya Hinohara, Manuel Reyes, Mahesh Ramchandani, Michael J. Reardon

**Affiliations:** a DeBakey Heart & Vascular Center, Houston Methodist Hospital, Houston, Texas, USA; b Texas A&M College of Medicine, Bryan, Texas, USA

**Keywords:** annular rupture, root enlargement, transcatheter aortic valve replacement, CPB, cardiopulmonary bypass, TAVR, transcatheter aortic valve replacement

## Abstract

We describe an 88-year-old woman who experienced annular rupture during transcatheter aortic valve replacement despite preventative measures. She underwent Y incision and rectangular patch for the double purpose of repairing the rupture and enlarging the aortic root. We highlight the heart team’s role in confronting this potentially catastrophic complication. (**Level of Difficulty: Advanced.**)

## Central Illustration

**Figure:**
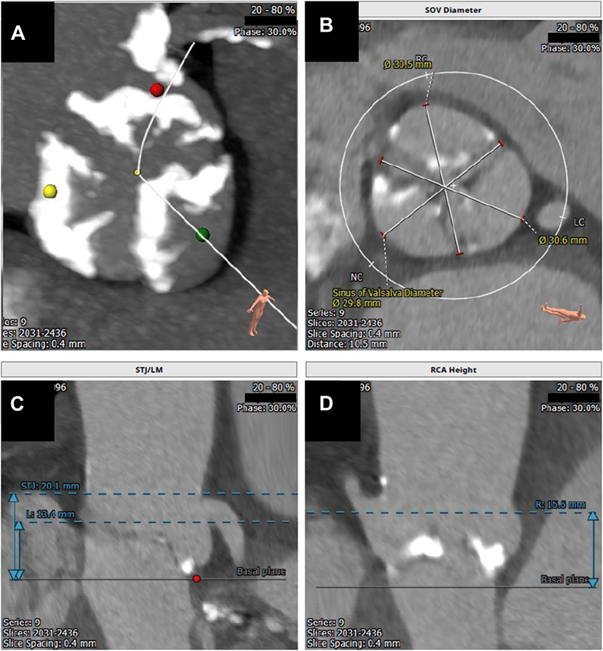

## History of Presentation

An 88-year-old woman presented with shortness of breath with minor activity and New York Heart Association functional class II symptoms. She had a 4/6 harsh systolic ejection murmur at the left sternal border.

**Learning Objectives**

• To describe the diagnosis and management of annular rupture during transcatheter aortic valve replacement by a heart team.
• To illustrate use of the Y annuloplasty surgical technique in allowing safe repair of annular rupture while avoiding full aortic root replacement.
• To emphasize that prompt and early intervention by an expert surgical team is paramount for a favorable outcome after annular rupture.

## Past Medical History

The patient had no significant past medical history.

## Investigations

**Figure 1:**
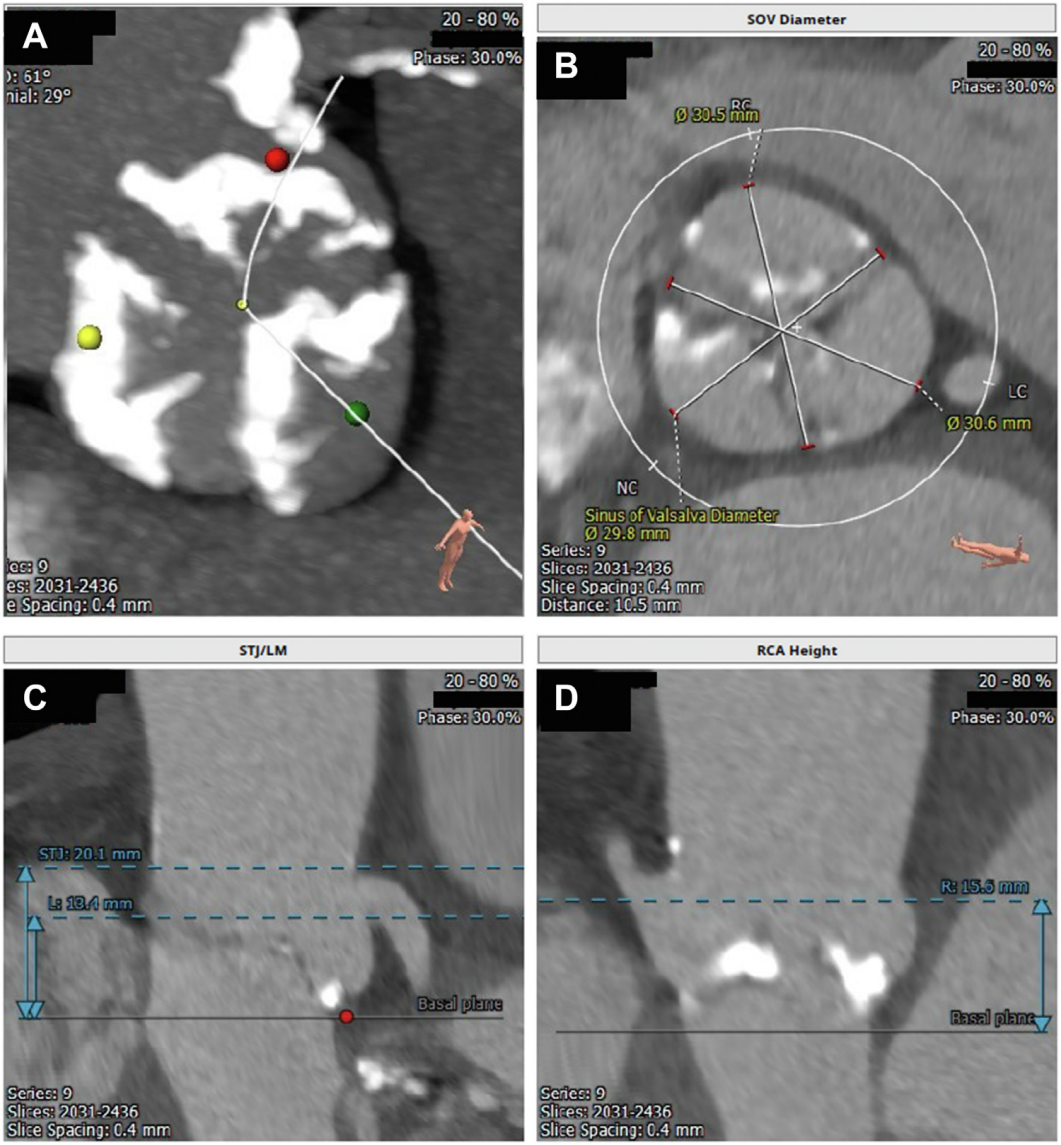
Calcified Transcatheter Aortic Valve The transcatheter aortic valve replacement protocol computed tomography showed a moderately calcified aortic valve and minimal ascending aortic calcification. LM = left main coronary artery; RCA = right coronary artery; SOV = sinus of Valsalva; STJ = sinotubular junction.

Echocardiography showed critical aortic stenosis (valve area: 0.7 cm^2^) with a mean gradient of 91 mm Hg, peak pressure gradient of 76 mm Hg, and Doppler velocity index of 0.19; the left ventricular ejection fraction was 65%. The transcatheter aortic valve replacement (TAVR) protocol computed tomography showed a native annulus area-derived mean diameter of 23.1 mm, annulus area of 427 mm^2^, left ventricular outflow tract area of 334 mm^2^, and a moderately calcified aortic valve and minimal ascending aortic calcification (Figure 1). Her STS score indicated a risk of mortality of 4.9%.

## Management

The patient was considered high risk for surgical aortic valve replacement and was scheduled for TAVR following shared decision making with the heart team.

In the hybrid operating room, a Sentinel embolic protection device (Boston Scientific) via the right radial artery was placed. Following initial aortogram (Video 1), preimplant balloon aortic valvuloplasty was conducted with a 20-mm True Balloon (Bard) and was well-tolerated (Video 2). A 23-mm Edwards Sapien 3 Ultra valve with nominal fill (Edwards Lifesciences LLC) was implanted using rapid ventricular pacing at 180 beats/min and pressure-controlled delivery at 6 atm with 5-second inflation to minimize the risk of rupture (Video 3). Positioning was confirmed via aortic root aortography (Video 4). Transthoracic echocardiogram confirmed excellent placement with no paravalvular leak.

Approximately 5 minutes after valve deployment, the patient developed severe hypotension. New pericardial effusion was recognized on transthoracic echocardiogram. Percutaneous pericardial access was obtained, and a drain was placed. Pericardiocentesis was performed, and more than 500 mL was taken from the pericardium and returned to the femoral vein. Blood pressure temporarily improved; however, the pericardial effusion persisted, and the oxygen saturation of the pericardial blood was systemic. It was recognized that the likely culprit of pericardial effusion/cardiac tamponade was arterial bleed from ventricular perforation or aortic root rupture. The patient was intubated, and a transesophageal echocardiogram probe was placed. Sternotomy was performed, and the pericardium was accessed; hemodynamics improved with release of the remaining tamponade. The patient was placed on cardiopulmonary bypass (CPB) via central cannulation, and the heart was decompressed immediately through the right superior pulmonary vein.

**Figure 2:**
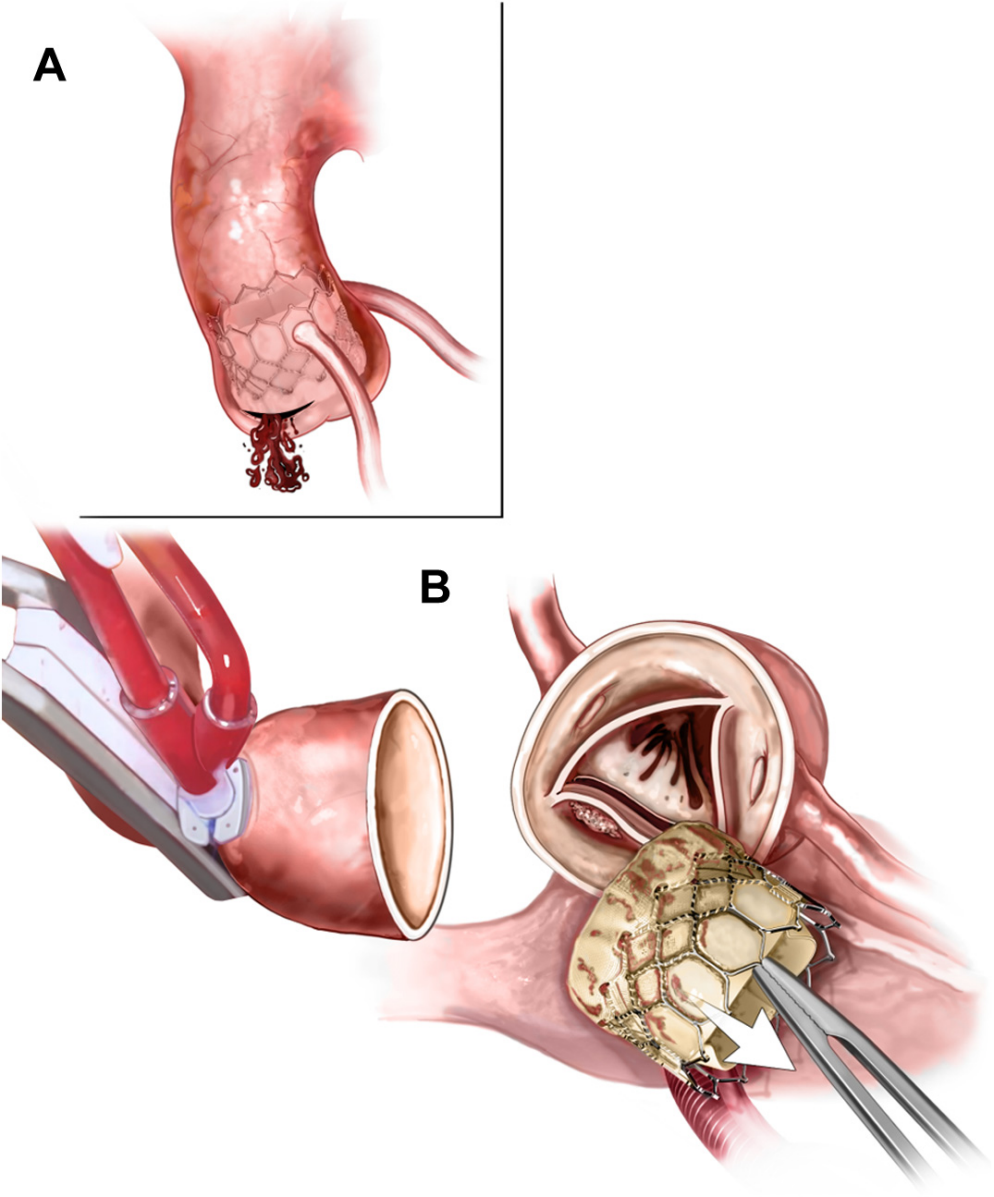
Subannular Rupture **(A)** Investigation of the aortic root revealed a rupture at the base of the noncoronary sinus where noncoronary cusp calcification pierced the annular wall. **(B)** The transcatheter aortic valve was explanted, and the native aortic valve was excised.

Hematoma and bleeding could be seen at the aortic root at the noncoronary cusp. After cross clamp, cardioplegia, and aortotomy, investigation of the aortic root revealed a rupture at the base of the noncoronary sinus where noncoronary cusp calcification pierced the annular wall. The transcatheter aortic valve was explanted, and the native aortic valve was excised (Figure 2).

**Figure 3:**
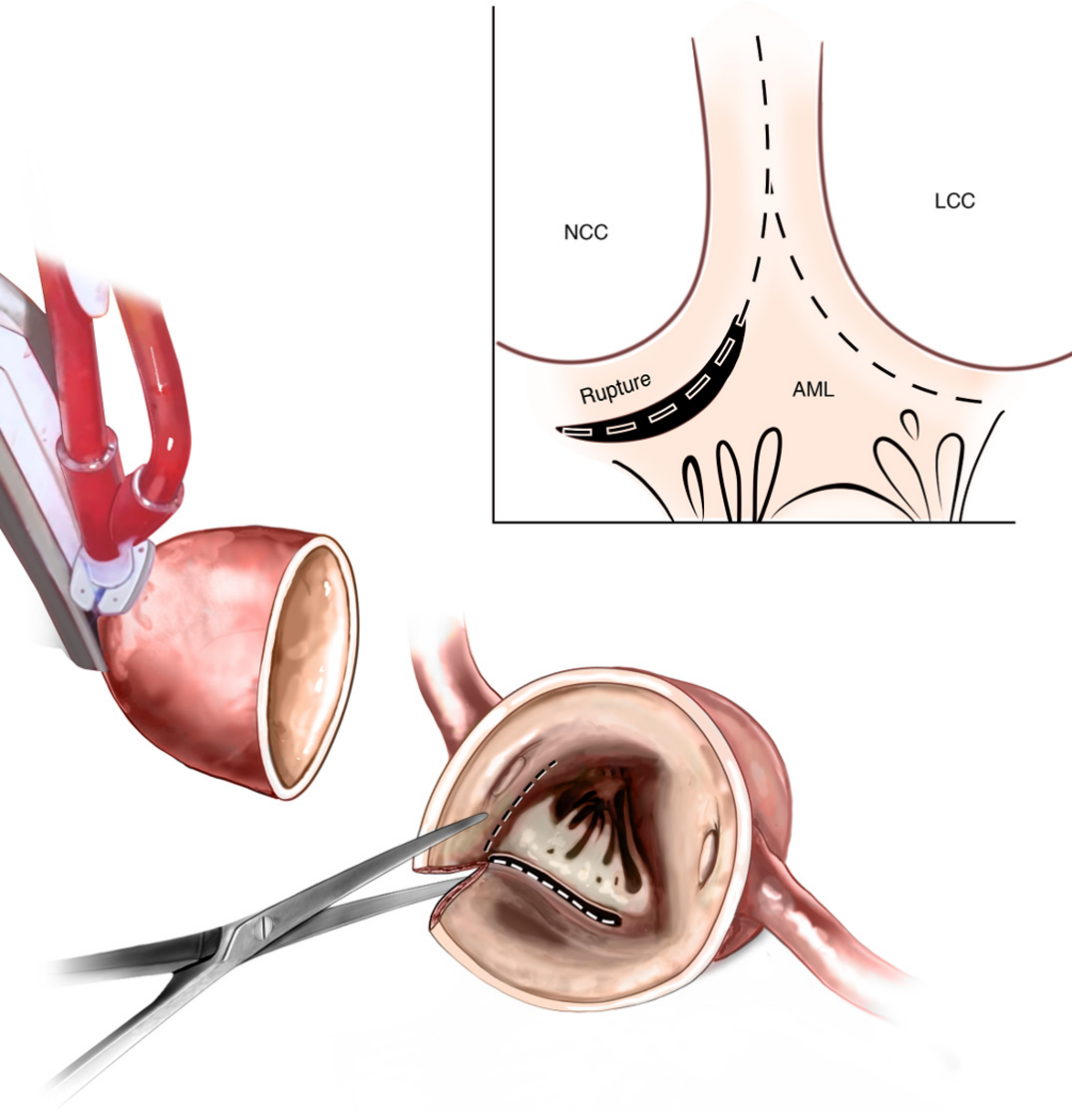
Annular Enlargement The incision was taken down to and through the junction of the left and noncoronary cusps and then diverted to the left coronary cusp to the nadir below the left main coronary artery. The annular disruption in the noncoronary cusp was used as part of the enlargement and, thus, extended until the membranous septum. AML = anterior mitral leaflet; LCC = left coronary cusp; NCC = noncoronary cusp.

**Figure 4:**
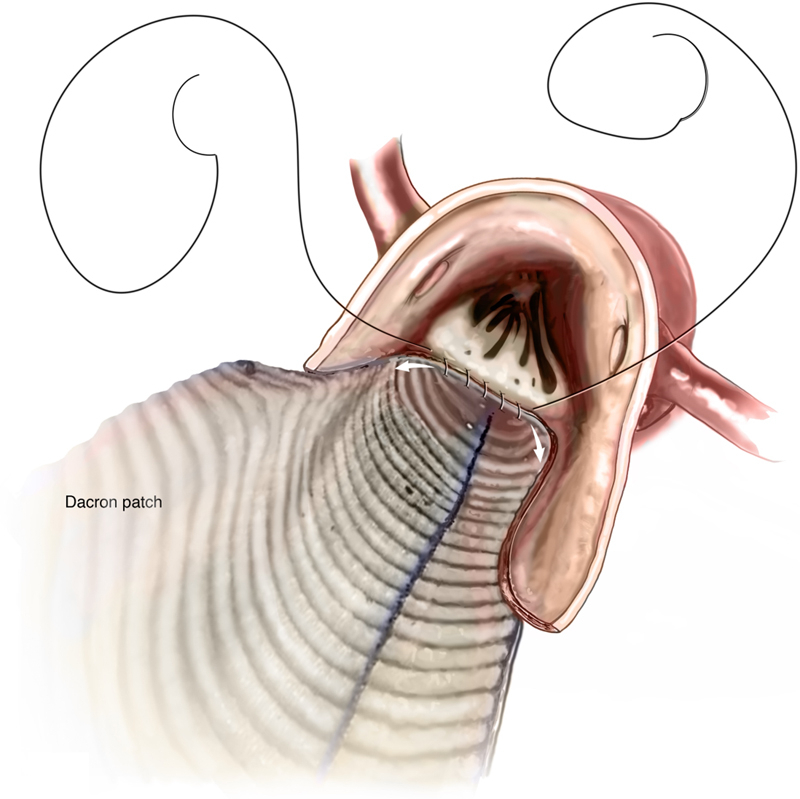
Graft Preparation A 30-mm synthetic polyester graft was split longitudinally, and a patch to match the width of the annular incision was fashioned. This patch was sewn in using 4-0 monofilament along the annular incision and up along the old annulus and aortic wall to above where the new valve would sit.

**Figure 5:**
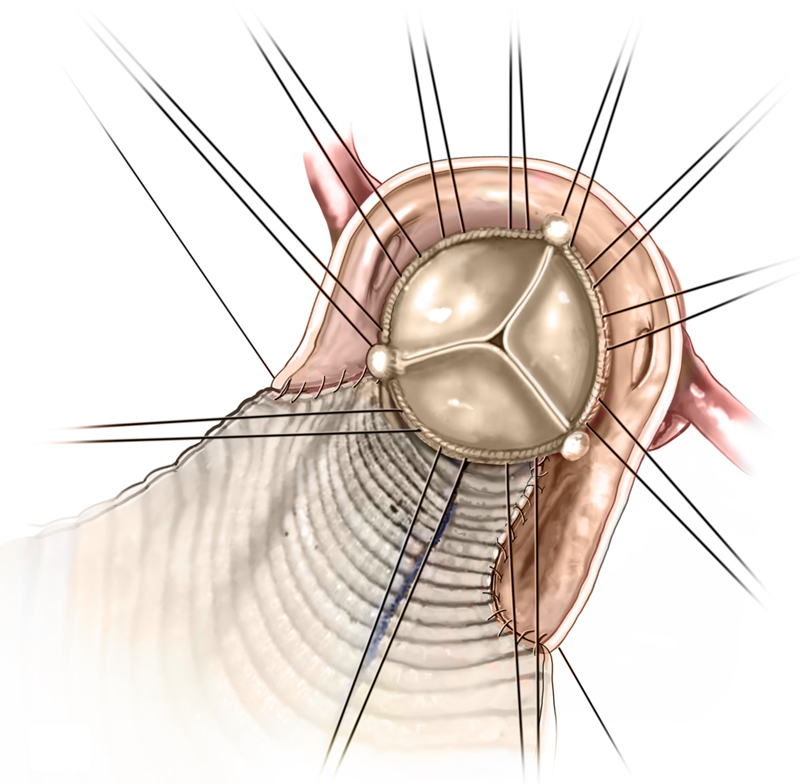
Valve Implantation A 25-mm bioprosthetic valve was placed.

The patient was barely sized to a 21-mm valve. Therefore, the incision was taken down to and through the junction of the left and noncoronary cusps and then diverted to the left coronary cusp to the nadir below the left main coronary artery. The annular disruption in the noncoronary cusp was used as part of the enlargement and, thus, extended until the membranous septum (Figure 3). A 30-mm Dacron graft (DuPont) was split longitudinally, and a patch to match the width of the annular incision was fashioned. This patch was sewn in using 4-0 Prolene (Ethicon, Inc) along the annular incision and up along the old annulus and aortic wall to above where the new valve would sit (Figure 4). The valve was then sized to 25 mm. A 25-mm Edwards Inspiris valve (Lifesciences LLC) was placed without difficulty (Figure 5).

**Figure 6:**
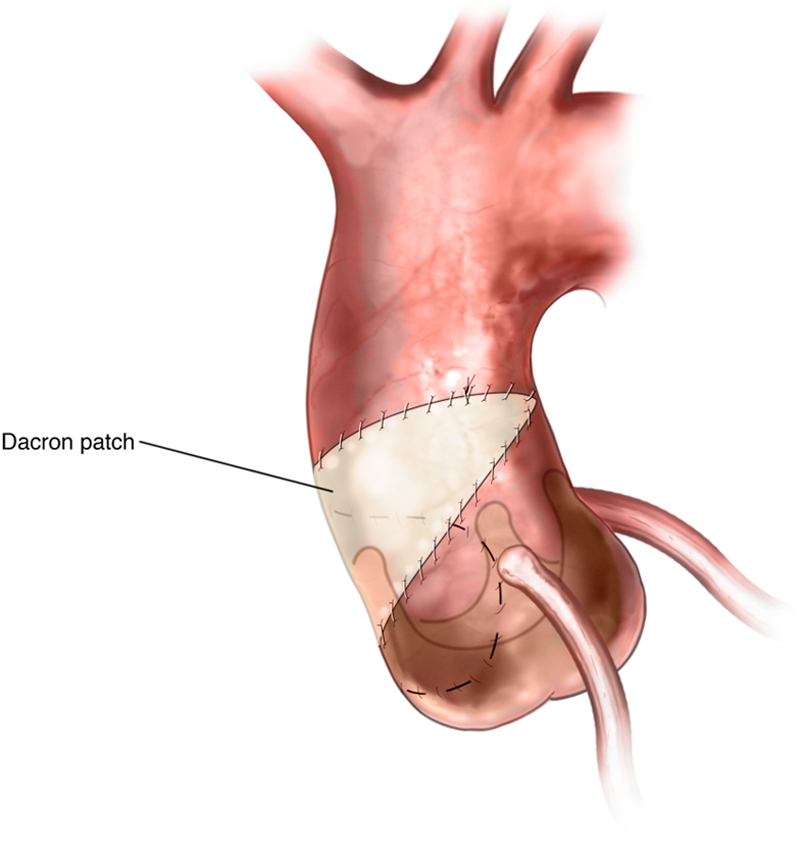
Patch The synthetic polyester graft was cut to fit the remaining opening of the aorta. The inferior and superior limbs were closed with running 4-0 monofilament.

The 4-0 sutures were then brought up and taken to the top of the aorta directly anteriorly on both sides. The graft was cut to fit the remaining opening of the aorta, and the inferior limb was closed with running 4-0 monofilament. The superior limb was closed with running 4-0 monofilament (Figure 6). After proper de-airing, the clamp was removed, and the patient was weaned from CPB. Postoperative echocardiogram showed a well-seated valve with no paravalvular leak.

The patient was extubated 4 hours after the procedure and transferred out of the intensive care unit on postoperative day 1. She was discharged home on postoperative day 8.

## Discussion

Annular rupture refers to injury occurring in the region of the aortic root and left ventricular outflow tract during TAVR.^1^ Early recognition and precise diagnosis of annular rupture are paramount to a successful outcome. Although treatment approaches may include conservative therapy or pericardial drainage, unstable patients will require surgical repair. The early initiation of CPB is paramount and requires efficient coordination among members of the heart team. This case illustrates the continuing need for a heart team approach with both interventional cardiologists and cardiac surgeons actively involved in the procedure.

Annular rupture with a balloon-expanded valve is fortunately rare, occurring in about 1% of all TAVR procedures, although subclinical occurrence of rupture renders the true incidence likely higher than reported.^1^ Aggressive oversizing of the valve may increase the risk for rupture but occurs only in the presence of 1 or more other factors, mainly calcification.^1^ Rupture is therefore not necessarily related to discrepancy between the native annulus size and the prosthesis.^1^ Possible risk factors for annular rupture include small aortic valve annulus; narrow aortic root; significant calcification in the aortic valve leaflets, even in a large aortic root; calcification of the annulus; calcium in the left ventricular outflow tract; calcification of the sinuses of Valsalva; heavily calcified bicuspid valve; severe asymmetric subaortic left ventricular hypertrophy; and global left ventricular hypertrophy in elderly, mostly female patients with decreased left ventricular compliance.^1^ In addition, studies have attempted to identify possible quantitative predictive factors of annular rupture, such as calcium score beyond the limit of safety or calcium nodules of more than 4 to 5 mm.^1^

Our patient demonstrated subannular rupture with a tear at the base of the noncoronary sinus, likely caused by balloon overdistension causing the cusp, containing bulky and calcified material, to press toward the calcified aortic wall during valve deployment.^1^ Some have suggested that limiting balloon expansion to 6 atm may help mitigate the chance of rupture.^2^ Although we limited balloon expansion to 6 atm, it was the pattern of calcification in the noncoronary cusp that allowed aortic wall perforation.

Techniques for repair of annular rupture include primary repair, patch annuloplasty, and aortic root replacement. In this case, the rupture of the base of the noncoronary cusp would not allow adequate primary repair. The surgical options were to patch the entire noncoronary cusp and place a small valve; to perform a full aortic root replacement; or to use the Y annuloplasty technique. The Y annuloplasty technique allowed us to safely repair the rupture while placing an adequately sized surgical valve and avoiding a full root replacement.^3^ We believe that both of these outcomes were of benefit to our patient, particularly given the growing agreement that implantation of larger valves allows for improved early hemodynamic performance and provides a substrate for potential future valve-in-valve if needed.^4^, ^5^, ^6^

## Follow-Up

The patient was doing well at the 2-month follow-up. Repeat echocardiogram showed good cardiac function and valve hemodynamics.

## Conclusions

Annular rupture is a rare but serious complication after TAVR. Identification of risk factors during preoperative planning is paramount. When free annular rupture occurs, accurate diagnosis and early conversion for surgical repair by an experienced heart team are crucial for a favorable outcome.

## Funding Support and Author Disclosures

Dr Reardon is a consultant to Medtronic, Boston Scientific, and Gore Medical. Dr Reyes is a consultant for Abbott, Edwards, and CSI. All other authors have reported that they have no relationships relevant to the contents of this paper to disclose.

## Appendix

Supplemental Video 1 Initial Aortogram Showing Calcified Aortic Valve (Coplanar View)

Supplemental Video 2 Preimplant Balloon Aortic Valvuloplasty

Supplemental Video 3 Transcatheter Aortic Valve Deployment With Pressure-Regulated Deployment Strategy

Supplemental Video 4 Final Aortography
